# Bloodhounds chasing the origin of blood cells

**DOI:** 10.1016/j.tcb.2025.03.003

**Published:** 2025-04-10

**Authors:** Lauren N. Randolph, Claudia Castiglioni, Manuela Tavian, Christopher M. Sturgeon, Andrea Ditadi

**Affiliations:** 1 San Raffaele Telethon Institute for Gene Therapy, IRCCS San Raffaele Scientific Institute, Milan, Italy; 2 Université de Strasbourg, Inserm, IRFAC / UMR-S1113, FHU ARRIMAGE, FMTS, Strasbourg, France; 3 Black Family Stem Cell Institute, Icahn School of Medicine at Mount Sinai, New York, NY; 4 Department of Cell, Developmental and Regenerative Biology, Icahn School of Medicine at Mount Sinai, New York, NY

**Keywords:** Hemogenesis, Hematopoietic stem cells, Hematopoietic development, Endothelial cells, Hemangioblast

## Abstract

The generation of blood cells during embryonic development involves a process resembling lineage reprogramming, where specialized cells within the vasculature become blood-forming, or hemogenic. These hemogenic cells undergo rapid transcriptional and morphological changes as they appear to switch from endothelial to blood identity. What controls this process and the exact nature of the hemogenic cells remains debated, with evidence supporting several hypotheses. This opinion paper synthesizes current knowledge and proposes a model reconciling conflicting observations, integrating evolutionary and mechanistic insights into blood cell emergence.

## The making of blood cells

The production of blood cells in adult organisms, a process named ***hematopoiesis***, follows for the most part an ordered hierarchical structure where blood stem cells (***hematopoietic stem cells***, HSCs) sit at the apex and generate the different types of blood cells. However, during embryonic development, the conceptus requires the simultaneous generation of both differentiated hematopoietic cells to meet the needs of the growing body and to coordinate the homeostasis of nascent tissues as well as HSCs and progenitor cells to establish and maintain a functional blood system throughout life. To meet these seemingly contradictory goals, the developing blood system is established via multiple, partially overlapping hematopoietic waves that give rise to a multilayered system (see Box 1). Curiously, blood cell production in the embryo is outsourced to a different lineage, with blood cells generated via a process that appears to be a «lineage reprogramming». In fact, all of the different waves of blood cell generation require the specification of a subset of cells that are integrated into the vasculature to become blood-forming, or ***hemogenic***. These cells represent a transient population that abruptly undergo transcriptional and morphological changes as they appear to switch from endothelial identity to blood. The exact nature of these specialized hemogenic cells and therefore the exact origin of blood cells remains controversial, with evidence supporting multiple hypotheses. Here the current knowledge and controversies about the lineage relationship between ***endothelial cells*** and the hematopoietic program are outlined and a model reconciling the different observations is proposed.

## In the beginning was the… hemangioblast

During vertebrate embryogenesis, the development of blood vessels and blood cells is coordinated to establish a functional circulatory system that provides nutrients and oxygen to all growing tissues. The intimate relationship between these two lineages was noticed more than a century ago with the observations of the physical association of blood cells with the endothelial lining of blood vessels in developing vertebrate embryos [1,2]. This close association between blood and endothelial cells, observed broadly across vertebrate embryos, was seen as evidence for the “hemogenic capacity of the young endothelium” [1]. As such, Jordan and Sabin hypothesized that blood cells may develop directly from endothelial cells, whose precursors Sabin called “angioblasts” [2] .

In contrast, Murray defined the mesoderm-derived cell “masses” located in the chick yolk sac (YS) where blood cells would later emerge as “***hemangioblasts***” [3], a cell population that gives rise to both blood and endothelial cells. A common clonal progenitor for these two lineages was hypothesized, but it wasn’t until 1998 that work with mouse embryonic stem cells demonstrated the existence of a clonal precursor cell with mesodermal origins that was capable of forming blood vessels and blood cells [4]. Of note, this is arguably the first and quintessential example of how work performed *in vitro* with embryonic stem cells gave fundamental insights into the early stages of embryonic development as only later it was shown that the hemangioblast was present also in the ***primitive streak*** of mouse embryos [5].

Given the complex onset of hemogenesis in the embryo, whether blood derives from a hemangioblast or hemogenic endothelial cells (HECs) is a recurrent controversy. A formal demonstration that blood progenitors are generated from an endothelial cell population was achieved through lineage tracing [6–8] and time-lapse imaging [9–13]. On the other hand, classical in vivo lineage tracing in the mouse embryo has failed to demonstrate a bipotent clonal contribution of hemangioblasts [14,15]. However, faithfully tracking these transient cells in vivo is challenging [16]. Only recently, a novel cellular barcoding approach demonstrated that the hemangioblast gives rise to both endothelial and blood lineages in vivo [17].This single cell lineage tracing demonstrated that along with the hemangioblast, other blood cell precursors can be captured that harbor a differentiation potential extending to other mesodermal lineages, including smooth muscle cells. This was already observed in both in vitro and ex vivo cultures [5,18] . We propose that these cells forming blast-like colonies in culture and displaying a broad mesodermal potential represent lateral mesoderm precursors that give rise to blood via the bipotent intermediate hemangioblast.

**Figure 1:**
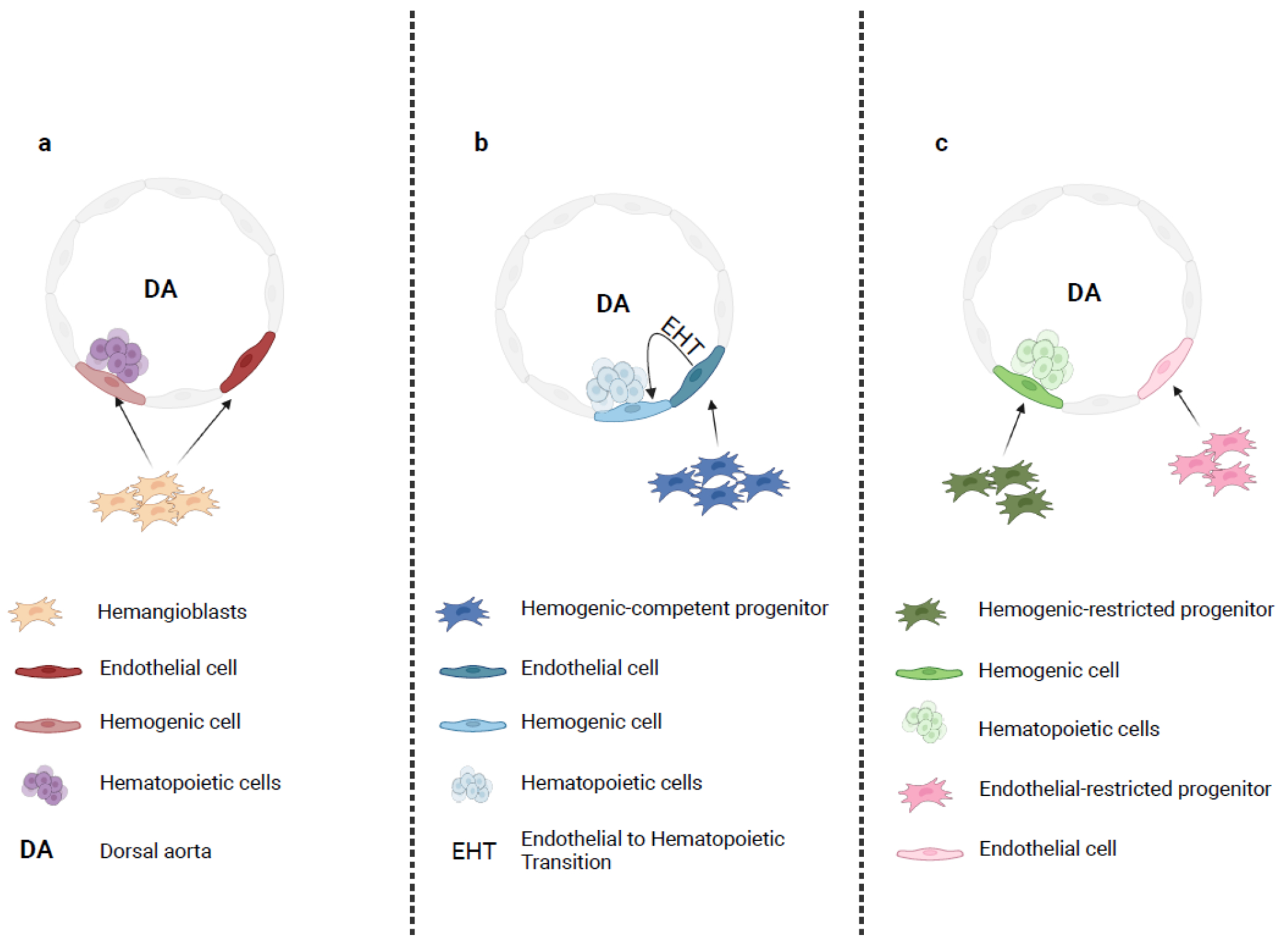
Models of intra-embryonic hematopoietic cells emergence. (a) The hemangioblast-based model: hemangioblasts migrating through the sub-aortic mesenchyme surrounding the ventral side of the dorsal aorta (DA) generate both endothelial and hemogenic cells. Hemogenic cells will then differentiate into blood cells. (b) The arterial-derived model: a hemogenic- competent progenitor gives rise to arterial endothelial cells that localize in the ventral side of the DA. These arterial cells undergo an endothelial to hematopoietic transition (EHT) resulting in hematopoietic cell formation. (c) Distinct lineages model: two separate precursors, the hemogenic-restricted progenitor and the endothelial-restricted progenitor, give rise to hemogenic cells and endothelial cells, respectively. This figure was created using BioRender (https://biorender.com/).

The hemangioblast and HECs are not necessarily the two parts of a dichotomy. In fact, a closer examination of the hemogenic progression of the hemangioblast derived from embryonic stem cells provided evidence supporting a comprehensive model in which the hemangioblast gives rise to blood cells through a hemogenic endothelial intermediate [19,20]. It is unclear whether this hemangioblast-based model applies to all the different hematopoietic programs of the developing embryo. Collectively the data indicate that all blood cells, including those belonging to the primitive program (see Box 1), are generated from HECs [19,21]. Historically a role for the hemangioblast has been restricted to the primitive hematopoietic program and indeed a HSC-competent hemangioblast has not been observed. Whether the hemangioblast is the precursor of the entire hemogenic endothelial lineage and therefore whether or not HSC-competent hemangioblasts exist are still open questions (Figure 1a). In vivo studies indicate that this might be the case [22], but these remain controversial as they employed modulation of Runx1 dosage which has profound impact on hematopoietic development [23]. In vitro evidence supports the model of a broader contribution of hemangioblasts to embryonic hemogenesis beyond the primitive program [24]. If a definitive hemangioblast exists, what is its developmental potential in vivo and where can it be found? When do endothelial and blood fates diverge? Studies examining single cells from mouse embryos or hPSC cultures demonstrate that hemogenic cells appear to be hematopoietic restricted as they predominantly produce blood cells, displaying little, if any, capacity to generate both blood and endothelial lineages [25–27]. It is possible that such a bipotent cell can be isolated earlier from the primitive streak, similar to precursors of the primitive hematopoietic program.

Alternatively, the hemangioblast could give rise to HECs only under the instruction of inductive local cues (Box 2). Functional studies in mice have demonstrated the presence of hematopoietic precursors not integrated in blood vessels in the ***subaortic patches***, located in the mesenchyme below the aortic floor [28]. Similar cells are present also in the subaortic mesenchyme in the human embryo, where they can be isolated as Angiotensin Converting Enzyme (ACE)+ cells starting from 24 days of gestation [29], i.e. before hematopoietic cells emerge from the wall of the dorsal aorta (DA; 27 days post-fertilization, [30]). While these studies support the existence of a putative hemangioblast in the subaortic mesenchyme which gives rise to blood cells via a HEC intermediate, further experiments are needed to show whether subaortic patches and ACE+ cells can generate both blood and endothelial cells at the clonal level. Of note, there is no evidence of a direct lineage relationship between subaortic mesenchymal cells and hemogenic cells. While this relationship cannot be excluded, the presence of hemogenic potential in subaortic patches in the mesenchyme could be due to the extensive tissue remodeling occurring at this developmental stage and incomplete migration of hemogenic cells to the intra-aortic niche.

## To be or not to be… an arterial cell

One of the most extensively researched questions is whether HECs originate from arteries or rather represent an independent lineage that uses arterial vessels as a niche for differentiation. Of note, this question seems to be mostly related to ***intra-embryonic*** hematopoietic development, because in the YS, hematopoietic progenitors emerge before and independently of proper arteriovenous specification [31]. However, new evidence shows that the nature of the vessel from which hematopoietic progenitors are generated impacts their potential [31], therefore a thorough characterization of the effects of arteriovenous specification perturbations on YS hematopoiesis is needed.

It is indisputable that blood cells arise from cells lining the lumen of major arteries and that the disruption of arterial specification in vivo leads to the severe impairment of hemogenesis in the DA, evidenced by an absence of hematopoietic clusters associated with the major arteries in most if not all arterial-defective mutants [32–37]. These observations laid the foundation for postulating an arterial origin of HECs: according to this hypothesis, arterial cells generate hematopoietic progeny through an endothelial-to-hematopoietic transition (Figure 1b) [12]. Supporting this model, recent papers indicate that the arterialization of endothelial cells generated from human pluripotent stem cells (hPSCs) leads to robust hematopoietic output and ultimately to HSC emergence in vitro [8,38]. However, the relationship between arterial and hematopoietic fate is complex, particularly when viewed from the perspective of NOTCH signaling. This pathway is the major driver of arterial identity, and perturbations to arterial specification ultimately have a negative effect on NOTCH signaling. Expression of genes associated with arterial identity, in particular Sox17 and Notch1, suppress the hematopoietic program, while repression of the arterial program, likely via Jag1- and/or Gpr183-mediated inhibition of NOTCH signaling, induces hemogenic potential [39–43]. The cell-autonomous requirement of NOTCH signaling for HSC specification, probably in multiple stages throughout their development, is well established [44–46]. However, the final stages of HSC maturation are accomplished in the absence of NOTCH signaling [47].

While the NOTCH signaling pathway is critical for both arterial and hemogenic fates, the mode, intensity, and length of its activation might vary between arterial and hematopoietic cell specification [44]. This raises the question of when and how this dynamic NOTCH signaling activation and subsequent inhibition is regulated locally in a portion of cells lining the lumen of the DA. Arterial cells are characterized by and require prolonged high levels of NOTCH signaling, unlike the transient and stage-specific requirements of HECs, with DLL4 instructing the arterial outcome and Jag1 needed solely for hemogenic cells [32,35,43,48–50]. For instance, mouse embryos lacking Jag1 show normal arterial development but a reduction in hematopoietic cells, indicating that Jag1 is required for the blood fate but dispensable for the arterial one and suggesting a role for distinct ligands in fate decisions [40]. Likewise, zebrafish studies showed that mutants for Jam1-Jam2, adhesion molecules that facilitate NOTCH signal transduction, are devoid of HSCs but display unaffected arterial identity [51]. At the same time, the induction of NOTCH signaling is sufficient to drive hemogenesis in venous vessels, albeit without activation of arterial genes [36,52]. All these observations are compatible either with a multistep process integrating an arterial specification followed by the initiation of the hemogenic program or with diverse origins of hemogenic and arterial cells (Figure 1b, c).

## Putting the pieces together

How can all of these seemingly contradictory data coexist? We propose here a potential model for the emergence of blood cells that encompasses these distinct observations.

First of all, we propose to clarify the nomenclature. The word endothelium refers to the tissue forming the layer of cells lining blood vessels [53]. As per this definition, endothelial cells make up the endothelium, but individual cells that only transiently line the lumen of a vessel do not necessarily become part of that tissue and hence should not be defined as contributing to the endothelium. Hemogenic cells, when lining the lumen of the vessels, do morphologically appear as squamous epithelial cells and express canonical endothelial markers (e.g. CD34, CD144, Tie2, etc.) along with RUNX1 [54–56]. But thus far all evidence shows that Runx1+ hemogenic cells become hematopoietic without asymmetric division, i.e. without leaving behind endothelial progeny [12]. In the absence of evidence of a persistent contribution of hemogenic cells to the endothelium beyond the transient localization in the lumen, we propose that hemogenic cells (HemCs), while simple, is a more appropriate term. Depending on their location and the embryonic stage, HemCs can express surface markers associated with arterial identity, such as CXCR4, DLL4, EFNB2, CD44, and CX40 [8,26,56–58]. As their expression is NOTCH-dependent [50,59–61], we believe these markers should be seen as the result of the local activation of this pathway triggered by surrounding niche cells, including arterial cells, rather than proper arterial specification. On the other hand, HemCs do not yet express hematopoietic markers such as CD41, CD43, CD45 etc., which should be looked at as maturation markers of HemCs as they undergo hematopoietic differentiation. CD117/cKit is another maker often used to identify HemCs. However, its expression in HemC appears to be context dependent as its expression is restricted to extra-embryonic cells, while intra-embryonically it marks cells in hematopoietic clusters [19,21,62].

From an evolutionary perspective, it is worth noting that hematopoietic cells predate the emergence of endothelial cells with the first phagocyte-like hemocytes detected in ***coelomic invertebrates***, such as Drosophila [63]. There is evidence indicating that, in invertebrates, hemocytes bud off the ***mesothelium*** in specialized domains, similarly to what was observed in the vertebrate DA [10–12,64,65]. It is interesting to note that in mammals there are two coeloms, both in regions associated with hematopoietic development: an extraembryonic one, the amniotic cavity in close spatial relation with the YS, and an intra-embryonic one, which on one side is lined by the ***splanchnopleura*** [66,67]. A direct lineage relationship between coelomic mesothelial cells and hemocytes has also been hypothesized based on their shared feature of ciliation. Indeed, primary cilia can be a potential evolutionary conserved marker to track hemogenic cells and their progeny, as cilia are present in vertebrate HemCs where they are required to coordinate NOTCH signaling [68,69]. In contrast, endothelial cells are only found in vertebrates, and as such emerged later in evolution than hematopoietic cells in response to the development of a closed circulatory system to address the needs of increasingly large and complex organisms [70]. Organisms across various phyla such as segmented worms, mollusks, echinoderms, and cephalochordates lack endothelial cells, but have specialized blood cells known as amoebocytes that can adhere to the vascular basement membrane [66,70]. In addition, amoebocytes in endothelium-less invertebrates can migrate and align in response to secreted VEGF and express mir126, a microRNA known for its role in regulating vascular homeostasis in vertebrates [71,72]. Altogether, these observations support an evolutionary emergence of endothelial cells from hematopoietic cells. Viewed from this lens, it would therefore seem unlikely that during evolution a complete reversal of the lineage relationship between blood and endothelium would occur, leading to a cell-autonomous requirement of endothelial identity for blood cell production. This evolutionary trajectory might explain why, despite functional divergence, hemogenic precursors and endothelial cells display convergent gene expression programs, according to the principle of genetic continuity described by Brenner[73]. When systems are connected by descent, genetic programs evolved by the predecessors are preserved and handed on to the progeny. The evolution of an endothelial-centric system for hemogenesis cannot be excluded; however, a linear progression from endothelial to hematopoietic fate would imply outsourcing the generation of cells performing essential and primordial tasks for multicellular organisms, like immune surveillance and oxygen transport, to a lineage whose primary function is profoundly divergent. Other additional factors need to be considered: first, in vitro and ex vivo studies show that HemCs are hematopoietic-restricted at a clonal level [26,27]. Additionally, a recent study revealed that blood cell emergence from committed human HemCs is linked to cell cycle re-entry [74], a process that developmentally is characteristic of cell differentiations [75] rather than lineage transitions, like that yielding neural crest cells [76] . Collectively, this suggests an early separation between endothelial and hemogenic lineages. This would imply that blood cells do not emerge via an endothelial-to-hematopoietic transition but rather by HemC differentiation, a process intimately connected to and supported by the surrounding niche that also contains endothelial cells.

**Figure 2:**
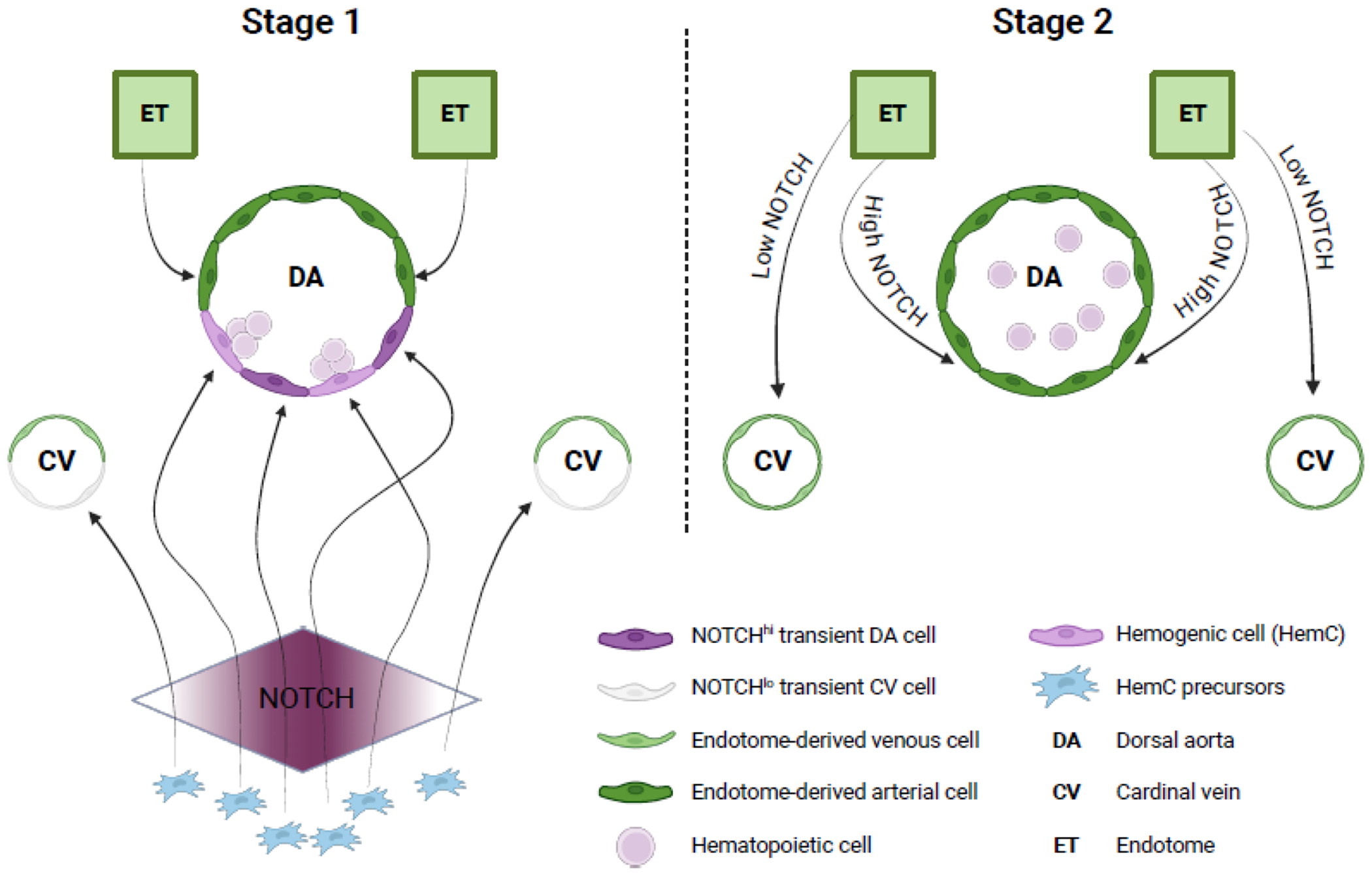
Proposed model of cell contributions to the DA during and after hemogenic events. There are distinct routes by which endothelial cells and hemogenic cells (HemCs) contribute to the dorsal aorta (DA). The first route consists of cells derived from the endotome (ET) that initially colonize only the dorsal side of the DA (Stage 1, left panel). The second route involves a separate lineage referred to as HemC precursors. All these HemC precursors harbor the potential of generating hematopoietic cells, but the final outcome is influenced by the levels of NOTCH signaling they detect during their migration. When they sense high levels of NOTCH, HemC precursors colonize the ventral layer of the DA, while low levels direct them to the ventral layer of the cardinal vein (CV)[99]. HemC precursors that detect intermediate levels of NOTCH signaling will colonize the DA and differentiate into hematopoietic cells. Later, (Stage 2, right panel) cells generated from HemC precursors are replaced in both the CV and DA by endothelial cells originating from the ET. The fate of these ET-derived endothelial cells is guided by the levels of NOTCH signaling: low levels support the venous fate in the CV, whereas high levels drive the arterial one in the DA [99]. This figure was created using BioRender (https://biorender.com/).

Based on these premises and focusing on the DA as an exemplary location for HemC development, we propose that there are separate routes to make endothelial and hematopoietic cells (Figure 2). Cells following one route can only contribute to the vasculature, like those of the ***endotome*** [67,77], and can become part of the inductive niche regulating blood cell emergence. Cells following the second route represent a separate lineage that colonizes specific sites, such as the ventral layer of the developing DA, and are, at least in potential, all HemCs. This is observed in the zebrafish and avian model, where the ventral layer of the developing DA is transiently composed almost uniquely of RUNX1+ cells. Notably, over time a near 100% conversion of cells lining the ventral side of the DA into hematopoietic cells is observed in zebrafish [12]. In addition, histological sections from mouse and human embryos showed that there are regions where the whole ventral side of the DA is RUNX1+ and therefore possibly composed entirely by HemCs (shown in mice: [78,79]; shown in human: [54,74]). In our model, cells form this second route, which transiently line the dorsal aorta, are later substituted by endothelial-restricted cells (of somitic origin, for what concerns the DA, as shown in the avian and zebrafish models [67,77]) during the extensive tissue remodeling phase occurring in the ***aorta-gonad-mesonephros*** region at those embryonic stages. Of note, the emergence of blood cells from HemCs is not a synchronous process, likely reflecting the timing of migration of HemC precursors to the DA anlage. The combination of NOTCH signaling sensed by these HemC-fated precursors (e.g. lower levels in aortic primordium, higher levels in mature DA [80,81]) and the local environment they encounter (e.g. the BMP4-producing subaortic mesenchyme, availability of retinoic acid [82,83]) will shape the hematopoietic potential of their progeny. This gradated exposure to key signals initiates the heterogeneous blood potential of HSCs and progenitors observed throughout life [84,85].

The model described above, centered on HemCs as transient cells of a hematopoietic-restricted lineage separated from endothelium and migrating through complex niches comprising arterial cells, assembles different pieces of the puzzle of developmental hematopoiesis and can explain 1) why some genetic mutants display only hematopoietic but not vascular defects; 2) why HemCs are observed only for a limited time; 3) why the time-specific activation of NOTCH signaling can turn venous cells into HemCs without activation of an arterial program; 4) why HemCs located in the same region generate progeny with heterogeneous hematopoietic potential.

## Concluding remarks

Such model needs to be thoroughly tested and some questions are still pending (see Outstanding questions box). First of all, time- and, possibly, space-specific cellular barcoding experiments, similar to those performed for the hemangioblasts [17], are needed to dissect the long-term contributions of HemCs and their precursors to arteries. The plasticity of the cells making up the ventral side of the mammalian DA needs to be thoroughly tested. Can they all be converted to blood? What happens to HemCs if RUNX1 is turned off? Some experiments performed in the zebrafish model have offered contrasting results: in one model cells died, in another they remained part of the aortic lumen [12,86]. In addition, there are several open questions that are not addressed by this model. For example, while long-term live imaging studies have not been performed yet in mouse embryos and cannot be performed in humans, all evidence gathered thus far indicates that, in these mammalian species, not all the cells on the ventral side of the DA will generate blood cells [62]. One possible explanation could be that mammalian HemCs necessitate tighter control of NOTCH signaling, likely mediated by lateral inhibition that results in one cell that is unique in a homogenous cell population [87,88]. This would explain the “salt and pepper” pattern of hematopoietic clusters in the arteries of the mammalian systems [62]. Alternatively, while all the ventral DA cells activate RUNX1 expression in response to mesenchyme-secreted BMP signaling, it is possible that the full hematopoietic program is initiated only in a fraction of the cells prior to the colonization of the DA, as studies using either Jam1-Jam2 mutants or Meis1 reporters suggest [51,89]. It remains to be assessed whether the cells following this HemC-fated route are hematopoietic restricted or whether they will become or have the potential to become endothelial cells. If so, it would mean that a definitive hemangioblast does exist.

**Figure 3:**
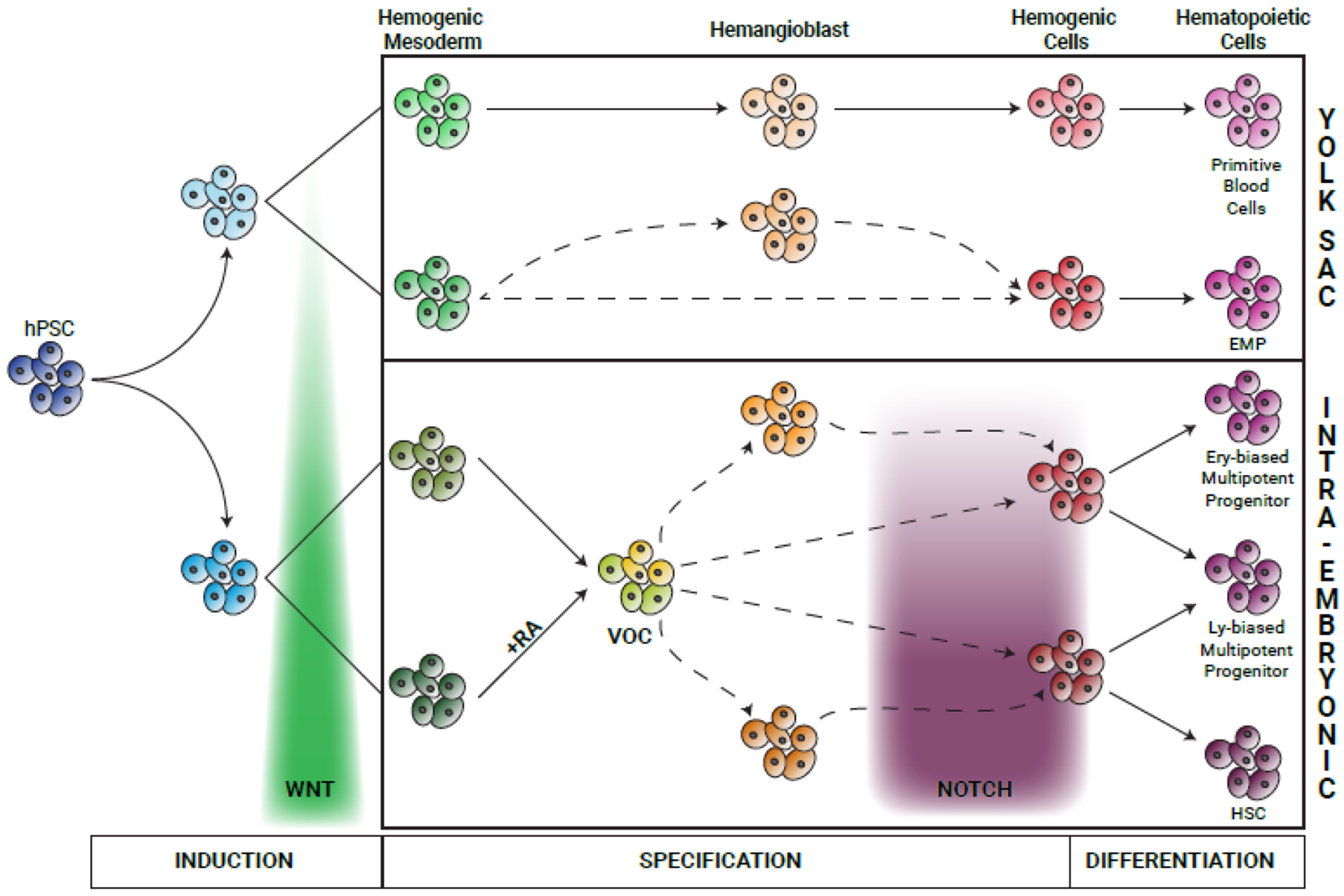
Proposed model for mammalian hematopoietic development. The schematic illustrates the proposed model for the induction and specification stages in hemogenic cell (HemC) emergence. The induction stage occurs over gastrulation and includes patterning of the different hemogenic mesoderms by varying exposure to Wnt signaling. HemCs contributing to YS-hematopoiesis are specified directly in the YS from two separate hemogenic mesoderms: one giving rise to HemCs harboring primitive hematopoietic potential via a hemangioblast intermediate, the other contributing to the erythro-myeloid progenitor (EMP)-restricted HemCs for which the presence of a hemangioblast has not been formally proven yet (dashed line). On the other hand, the specification of intra-embryonic HemCs occurs notably at the vessel of confluence (VOC) and may or may not include a hemangioblast stage, as indicated by dashed lines. Different levels of exposure to NOTCH signaling during migration from and remodeling of the VOC results in the varied hematopoietic differentiation potential of HemCs in the embryo.

If HemCs represent a separate lineage, when and where are they specified? This will be another important area to study in the future as little data is currently available and we can only offer some speculation. It must be noted that the very first RUNX1+ cells, which we can assume are all fated to become hematopoietic, are observed in the mouse embryo as early as E7.5, forming a structure called the “***vessel of confluence***” (VOC) [90]. The VOC is found at the base of the allantois where the neo-formed umbilical vasculature amalgamates with the embryonic DA and the vitelline (also named omphalomesenteric) artery. All these arteries are active hemogenic sites and harbor the majority of intra-arterial hematopoietic clusters [31,91]. Indeed, in the human embryo most of the CD32+ committed HemCs line the lumen of the arteries at the bifurcation of the DA and vitelline artery [74]. Given the extensive remodeling that occurs in that region [90–92], it is entirely plausible that all HemCs observed in these major arteries are derived from cells specified in the VOC that later are distributed to the different vessels where they continue their differentiation. If this is proven correct, we could imagine a scenario where it is during ***gastrulation*** that all the hematopoietic programs are induced yielding two separate mesodermal populations (Figure 3): one destined to contribute to all YS hematopoiesis (see Box 1), including the primitive hemangioblast, and one contributing to the VOC where HemCs found intra-embryonically as well as in umbilical and vitelline arteries will be specified. It is worth noting that the region at the base of the allantois already acts as the specification niche for primordial germ cells that also later migrate to the same aorta-gonad mesonephros region [93]. This pattern of hematopoietic induction followed by specification is indeed recapitulated in vitro by differentiating hPSCs. In this model, HemC population can be discreetly traced to distinct mesodermal populations [94–97], which can be referred to as hemogenic mesoderms (HMs). The distinct HMs are induced concomitantly, with the one contributing to YS hematopoiesis (encompassing both the primitive and erythro-myeloid progenitor programs) and segregating to a CD235+ population [94], while HemCs expressing HOXA genes indicative of an intra-embryonic allocation track back to CD235neg HM [94,95]. Based on the gradient of WNT signaling sensed, some of these CD235^neg^ HM cells become competent to respond to retinoic acid signaling and are poised to become HSCs [38,95]. These distinct HM cells can be also identified in the gastrulating CS7 human embryo [95,98]. Therefore, we speculate that hematopoietic lineages are induced during gastrulation, which would represent the stage where the blood and endothelial lineages diverge.

Careful dissection of these events using ad hoc lineage tracing strategies that ideally integrate space and time, combined with multi-omic analysis in animal models and the molecular dissection of early embryonic stages using hPSCs will illuminate and bring clarity to the complex process of the establishment of the hematopoietic system. Since each hematopoietic developmental program yields cells that harbor clinical potential, resolving how blood generation unfolds in the embryo will ultimately guide the efforts to derive specific clinically relevant cells in vitro.

## Box 1 – Hematopoietic programs during embryonic development

The establishment of the blood system during embryonic development is a complex and multifaceted process characterized by the interplay of various hematopoietic programs and highlights evolutionary adaptations that shaped hematopoietic cell function (Box 1 Figure). Primitive hematopoiesis is the first wave of blood cell production that occurs in the yolk sac (YS) around embryonic day (E) 7.5 in mice. This program is responsible for generating blood cells that resemble those found in ancient species, such as hemocytes in insects [100]. The hallmark characteristics of primitive hematopoiesis include its limited duration and potential, primarily producing erythrocytes as well as macrophages, and megakaryocytes. All these hematopoietic cells exhibit properties distinct from their post-natal counterparts [101–103]. Notably, while the emergence of hematopoietic progenitors belonging to this program occurs during a narrow window of time (around 24 hours in mice), lineage tracing studies demonstrated that this “wave” generates microglial cells, the professional phagocytes of the central nervous system, that persist through adulthood [104]. During this primitive phase, erythrocytes serve a critical function in oxygen transport within the developing embryo. They produce embryonic hemoglobin with an affinity suited for the hypoxic environment of the womb. As the embryo grows and requires more efficient oxygen delivery, a vascular system develops that connects the YS to the embryo proper, facilitating the circulation of these primitive cells to various tissues [103].

Following primitive hematopoiesis, cells with multi-lineage potential emerge, first identified in the YS as erythroid-myeloid progenitors (EMPs). These progenitors have a broader differentiation potential compared to their primitive counterparts, capable of generating not only erythrocytes and megakaryocytes but also a wider array of myeloid cells such as granulocytes [105]. Interestingly, while EMPs are primarily associated with myeloid lineage development and erythroid cells expressing fetal isoforms of hemoglobin, they also possess limited potential to give rise to T- and NK-lymphoid cells, with rare multipotent progenitors (MPPs) identified in the EMP fraction [106] . However, they do not generate B-cells or hematopoietic stem cells (HSCs) [105,106]. EMPs play a vital role in shaping the adult immune system as they give rise to most tissue-resident macrophages, with the exclusion of microglia which has been mostly traced to primitive hematopoiesis [107,108]. This distinction underscores the evolutionary significance of different progenitor populations in establishing immune cell diversity.

As development progresses, intra-embryonic MPPs, capable of differentiating in all blood cell lineages but devoid of repopulating ability when transplanted, arise before bona fide hematopoietic stem cells (HSCs) can be detected. Recent studies have demonstrated that these embryonic HSC-independent MPPs not only sustain the production of blood cells during the fetal stages of embryonic development but also contribute significantly to postnatal hematopoiesis [109,110]. HSCs are specified in the AGM region at E10.5 in the mouse embryo [111]. These cells possess unique properties, including selfrenewal, which allows them to produce identical daughter cells without differentiation, and the capacity to generate all adult blood lineages throughout an individual’s life.

Human developmental hematopoiesis has been less extensively studied and the limited availability of human embryonic tissue makes studying the early phases of the establishment of the blood system particularly challenging. However, collectively all human embryo studies, reinvigorated by the recent advances in single-cell technologies that enabled the identification of rare populations based on their molecular profile, point at a conserved layered architecture. Primitive hematopoietic progenitors can be detected in the pre-circulation YS at Carnegie stages (CS) 7-8 of human development (16-18.5 days after fertilization) [98,112–114] where they generate erythroblasts expressing embryonic ζ/ε globins [115,116]. The second hematopoietic wave has not been well-defined in human embryos as it is not possible to faithfully track the emergence of different hematopoietic progenitors. However, scRNA-seq analysis helped identify early human YS–derived myeloid progenitors that seed the liver by CS12 [117]. CS12 human YS also contain innate lymphoid-biased multipotent progenitors that emerge prior to hematopoietic stem cell formation [118]. Collectively these progenitors likely represent the human correlative of the murine YS-derived EMP program. Of note, scRNA-seq analysis of CS10 embryos also helped detect intraembryonic hematopoietic progenitors that are similar to those found in the YS at CS11 and might therefore represent intra-embryonic MPPs. The first human multilineage hematopoietic repopulating cells develop intra-embryonically, associated with the ventral domain of the dorsal aorta [119–121], and therefore follow a pathway that is highly conserved across vertebrate species.

Studies using human pluripotent stem cells (hPSCs, which include both embryonic stem cells as well as induced pluripotent stem cells) have also brought strong support to the hypothesis that the human hematopoietic system is built in multiple waves. In particular, hPSC studies showed that it is possible to obtain specifically each of the different hematopoietic programs using stage-specific manipulation of regulators such as BMP, Activin/TGFb, WNT, NOTCH, and retinoic acid (RA) signaling. Briefly, through a WNT-independent (WNTi) process, hPSCs differentiate rapidly towards either a CD43+ population of primitive haematopoietic progenitor cells (HPCs) or a CD34+ hemogenic population that displays all the hallmarks of YS-like hematopoiesis [94,96]. Conversely, in a WNT-dependent (WNTd) process, hPSCs give rise to HOXA+ hemogenic cells with NOTCH-dependent, clonal multi-lineage potential, indicative of intra-embryonic-like hematopoiesis [26,94,97]. However, if these cells are derived under RA-independent (RAi) conditions, they lack any observable multilineage engraftment potential and require the expression of multiple transgenes for HSC-like function [122]. Nevertheless, when this same population is exposed to a stage-specific RA signaling, it will result in the generation of hemogenic cells that in turn give rise to hematopoietic cells with robust multilineage potential both in vitro and in vivo, including HSC-like cells [38,95].

## Box 2 – The HSC inductive niche

Studies conducted in various animal models have highlighted the supportive role of the tissues surrounding the areas where HSCs are generated in vertebrates that act as an inductive niche. These studies, based on transplants, chimeras and lineage tracing, have shown that interaction between hematopoietic precursors and the surrounding instructive tissues is essential for HSC induction. The subaortic mesenchyme, surrounding the ventral side of the dorsal aorta, plays a critical role in HSC specification, with the co-culture of HSC precursors with ventral stromal cells supporting the full maturation and formation of HSCs [123]. Lineage tracing studies have shown that both the dorsal aorta and surrounding ventral mesenchyme have a splanchnopleural origin [48,124,125]. However, active remodeling is underway at those developmental stages and other cells with different origins contribute to the HSC specification niche [77,126]. For example, as the surrounding mesenchyme is remodeled, lineage tracing studies suggest the presence of two PDGFR+ mesenchymal populations in the AGM region, one derived from the mesoderm and the other from the neural crest [126], the latter colonizing the region at the end of the hemogenic stage. These studies show that mesoderm-derived cells are those supporting the emergence of the HSC program, with little if any contribution from the neural crest-derived mesenchyme. However, in zebrafish loss of neural crest cells or inhibition of their migration severely perturbed HSC development, indicating that neural crest cells also play a role in the HSC inductive niche [127,128]. This might be due to catecholamine-secreting neural crest-derived sympathoadrenal cells surrounding the DA that in mice seem to regulate HSC numbers, affecting HSC maintenance after their emergence rather than specification [129,130].

Like the subaortic mesenchyme, vascular smooth muscle cells (VSMCs), originating from diverse tissues like the neural crest, splanchnic mesoderm, and somites, are also in direct contact with the dorsal aorta, and their differentiation occurs simultaneously with HSC emergence [124,131]. In chick embryos, VSMC marker expression begins on the ventral side of the aorta, the site of HSC origination [132]. Signaling pathways like NOTCH and PDGF, crucial for HSC specification, also regulate VSMC differentiation [133,134], suggesting VSMCs or their precursors contribute to the niche that induces HSC formation. Other niche tissues, such as the gut, somite, and urogenital ridges, contribute to HSC specification with long range signals or indirectly [135,136]. Primitive myeloid cells (see Box 1) may also support early HSC development [137,138].

How the niche controls HSC emergence is subject of intense study. BMP signaling is critical for early mesodermal development and the dorsoventral patterning of the AGM niche [139]. BMP signaling directly coordinates the specification of HECs, as BMP is essential to induce Runx1 expression [48,83] in HSC precursors. However, BMP signaling is tightly controlled and its downregulation is required later for full HSC maturation. This is achieved through the secretion of the BMP inhibitors NOGGIN and BMPER from the inductive niche [135,140]. Another signaling pathway with an iterative role is FGF, which was shown to be essential for mesoderm formation and interacts with Wnt16/Notch pathways in the niche to support the effective HSC specification [141,142].

Other secreted signals, such as SHH/IHH, as well as inflammatory signals have a positive regulatory influence on HSC emergence [136,143]. For example, interleukin-1, interleukin-3, interferons, prostaglandin E2 (PGE2), and TNFa are each required for optimal specification of HSCs in mouse and zebrafish where they can also expand HSC numbers [137,144–147]. Notably, inflammatory signals seem to play a role in other lineage conversions that do not yield hematopoietic output [148]. This implies that they regulate biological processes at the basis of change of identity, likely directing the transcriptomic and epigenetic remodeling as occurs in other settings [149]. However, the cellular source of these inputs has not been determined. Primitive myeloid cells interact with emerging and maturing HSCs in both the specification and maturation niches so they can act as a possible source of inflammatory signals, as they were shown to be a source of critical IFNb and IFNg as well as matrix metalloproteases 2 and 9 (Mmp2, Mmp9) required for the release of HSCs from their specification niche [138]. Alternatively, it has been suggested that activation of inflammatory pathways can occur as a consequence of the reactivation of endogenous transposable elements directly in HemCs [150].

## Outstanding questions

- Do HemCs contribute to arterial endothelium? If so, for how long?
- How many hemogenic mesodermal populations are there?
- Does an HSC-fated hemangioblast exist?
- How is the differential regulation of NOTCH signaling between arterial and hemogenic endothelial cells controlled? What are the specific targets for the specification of each lineage?
- When and how do the hematopoietic and endothelial programs diverge during development?
- How plastic are HemCs?

## Highlights

- During embryonic development the establishment of the hematopoietic system is associated with that of endothelial cells, as blood cells appear to emerge first from cells lining the blood vessels.
- Major arteries of the developing embryo, such as the dorsal aorta as well as the vitelline and umbilical arteries, are the sites of the emergence of the progenitor pool of hematopoietic cells that sustain blood cell production throughout life.
- Arterial and nascent hematopoietic cells display shared expression of cell surface markers and transcription factors.
- The exact relationship between blood cells and arteries is still unresolved due to contrasting evidence.

## Glossary

Hematopoiesis: Hematopoiesis is the biological process of generating blood cells from hematopoietic stem and progenitor cells.
Hematopoietic Stem Cells (HSCs): Hematopoietic stem cells (HSCs) are specialized stem cells primarily found in the bone marrow that can differentiate into all types of blood cells as well as selfrenew to guarantee the homeostasis of the hematopoietic system.
Hemogenesis: Hemogenesis refers to the process of blood cell production from non-hematopoietic cell types.
Endothelial cells: Endothelial cells are specialized cells with squamous epithelial morphology that make up the endothelium and line the interior surfaces of blood vessels and lymphatic vessels, playing a crucial role in regulating blood flow, permeability, and immune response.
Hemangioblast: Hemangioblast is defined as a bipotential precursor that at a clonal level is capable of differentiating into both hematopoietic and endothelial cells.
Primitive streak: Primitive streak is a dorsal structure that forms in the epiblast of early vertebrate embryos. It establishes bilateral symmetry and is associated with the onset of gastrulation, leading to the formation of the three germ layers. As epiblast cells move through the primitive streak, they differentiate into either mesoderm or endoderm.
Subaortic patches: Subaortic patches in embryonic development are localized structures that form beneath the aortic region and do not contain endothelial cells. Notably, these patches contain cells expressing markers of hematopoietic precursors that may have repopulation capacity.
Intra-embryonic: Intra-embryonic refers to structures, tissues, or processes that occur within the developing embryo itself and excludes extra-embryonic tissues such as the yolk sac, amnion, chorio, placenta, etc.
Coelomic invertebrates: Coelomic invertebrates are animals that have a true coelom, a fluid-filled body cavity fully lined by mesothelium. This group encompasses various phyla including annelids, mollusks and arthropods.
Mesothelium: Mesothelium is a simple squamous epithelium that lines the body’s serous cavities and internal organs, forming the epithelial layer of serous membranes. It plays a key role in producing lubricating fluid, allowing organs to move smoothly against each other.
Splanchnopleura: Splanchnopleura is a layer of tissue in the embryo formed from the inner layer of mesoderm and the underlying endoderm, which develops into the embryonic viscera.
Endotome: Endotome refers to a specific endothelial precursor population, which arises within a subcompartment of the somite. It has been first defined in zebrafish and the transcriptomically identified in mouse embryos.
Aorta-Gonad-Mesonephros (AGM): A region located in the posterior part of the embryo, which includes the developing aorta, genital ridges, and mesonephros, that is responsible for the formation of hematopoietic stem cells.
Vessel of confluence (VOC): Conserved among placental mammals, the vessel of confluence (VOC) is the location where the primary arterial blood vessels of the conceptus converge, including the embryonic dorsal aorta, umbilical artery, and vitelline artery.
Gastrulation: Gastrulation is the early embryonic process during which a single-layered blastula reorganizes into a multi-layered structure called the gastrula, establishing the foundational germ layers that will develop into various tissues and organs.
